# Supplementary material for: Tau drives cell specific functional isolation of the hippocampal formation
Source: bioRxiv. 2025 Aug 11:2025.08.10.669580. Preprint. [Version 1] doi: 10.1101/2025.08.10.669580 (PMC12363901; doi:10.1101/2025.08.10.669580)
Supplement: Supplement 1 [file NIHPP2025.08.10.669580v1-supplement-1.pdf]

bioRxiv preprint doi: https://doi.org/10.1101/2025.08.10.669580; this version posted August 11, 2025. The copyright holder for this preprint (which was not certified by peer review) is the author/funder, who has granted bioRxiv a license to display the preprint in perpetuity. It is made available under aCC-BY 4.0 International license.

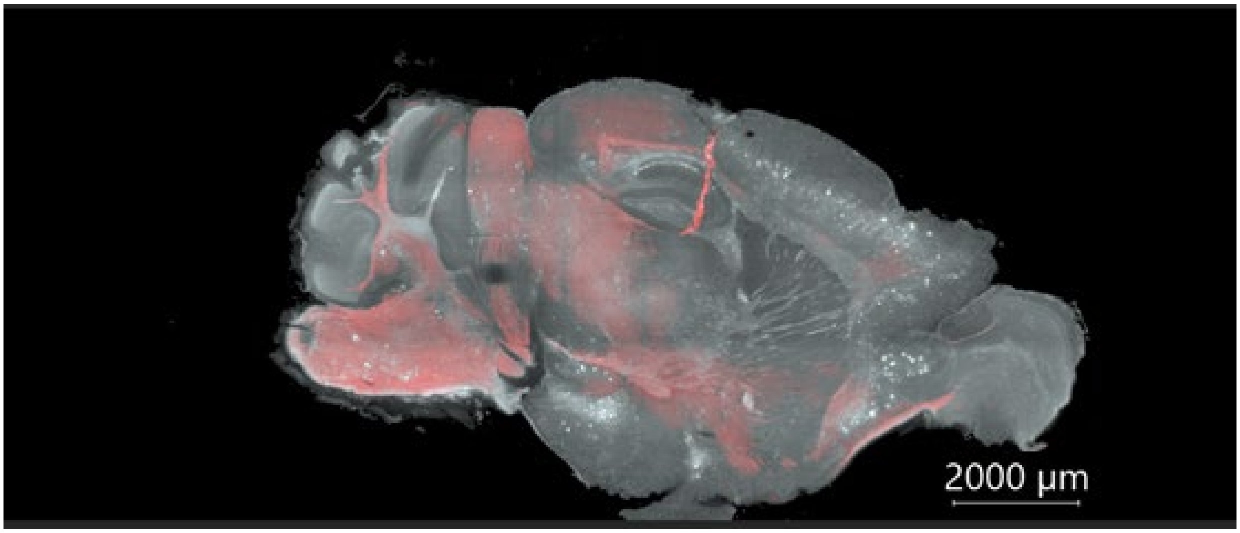

Supplemental Figure 1. Location of electronics (red) implanted in the hippocampus of a ThyTau22 mouse stained with AT8 (white).

bioRxiv preprint doi: https://doi.org/10.1101/2025.08.10.669580; this version posted August 11, 2025. The copyright holder for this preprint (which was not certified by peer review) is the author/funder, who has granted bioRxiv a license to display the preprint in perpetuity. It is made available under aCC-BY 4.0 International license.

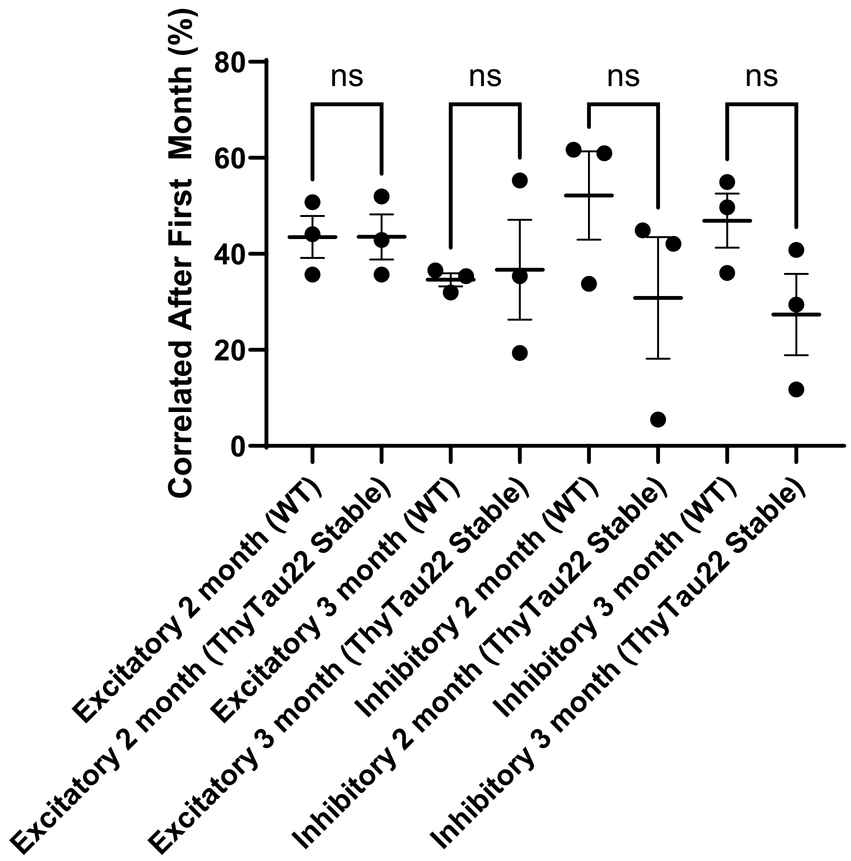

Supplemental Figure 2. No difference in the stability of correlated neuron pairs after 2- or 3-months of recording when comparing WT and ThyTau22 stable-firing neurons.

bioRxiv preprint doi: https://doi.org/10.1101/2025.08.10.669580; this version posted August 11, 2025. The copyright holder for this preprint (which was not certified by peer review) is the author/funder, who has granted bioRxiv a license to display the preprint in perpetuity. It is made available under aCC-BY 4.0 International license.

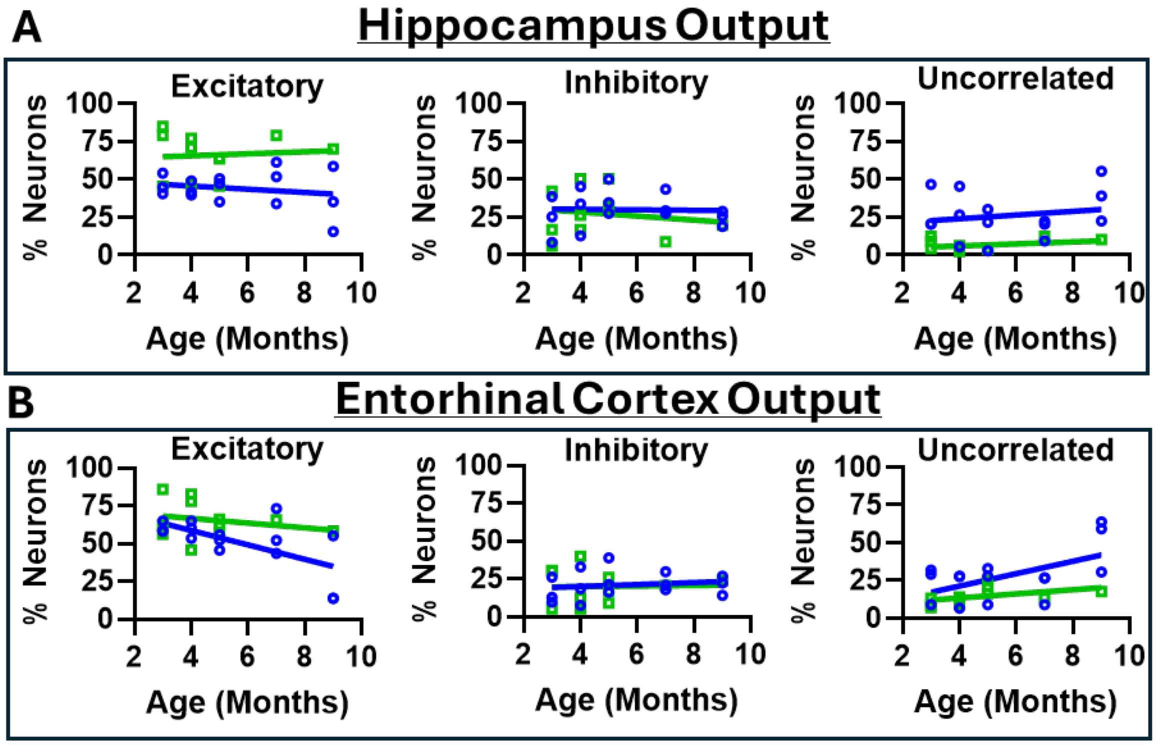

Supplemental Figure 3. The number of neurons with excitatory output decreases over time in both the hippocampus and entorhinal cortex in Thy-Tau22 (blue) but not WT (green) mice. The number of neurons with inhibitory output do not change over time for either Thy-Tau22 or WT mice. Each shape represents the proportion of neurons from an individual mouse (n=3 WT, 3 ThyTau22). Values were normalized as a percentage of all neurons recorded in each individual mouse. These data show neurons with excitatory correlations are becoming uncorrelated rather than permanently inactivating or dying.

bioRxiv preprint doi: https://doi.org/10.1101/2025.08.10.669580; this version posted August 11, 2025. The copyright holder for this preprint (which was not certified by peer review) is the author/funder, who has granted bioRxiv a license to display the preprint in perpetuity. It is made available under aCC-BY 4.0 International license.

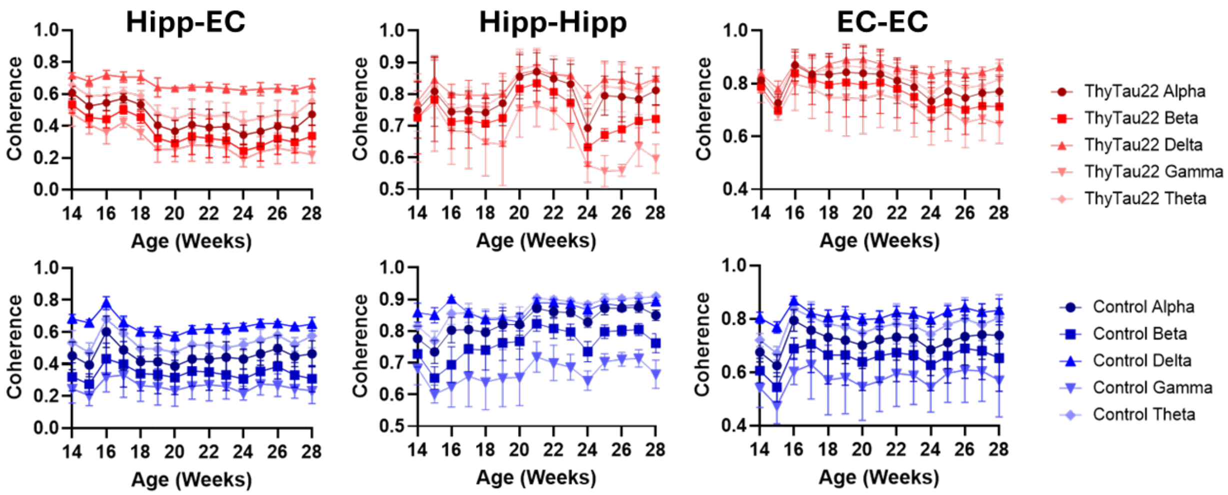

Supplemental Figure 4. Average change in coherence across mice (n=3 WT, 3 ThyTau22) for different filtered frequency bands
